# Distribution, Source Apportionment and Risk Assessment of Phthalate Esters in the Overlying Water of Baiyang Lake, China

**DOI:** 10.3390/ijerph20042918

**Published:** 2023-02-07

**Authors:** Chang Liu, Liguo Fu, Hui Du, Yaxue Sun, Yihong Wu, Cheng Li, Jikun Tong, Shuxuan Liang

**Affiliations:** 1 Key Laboratory of Hebei Provincial Analytical Science and Technology, College of Chemistry and Environmental Science, Hebei University, Baoding 071002, China; 2 Hebei Provincial Academy of Ecological Environmental Science, Shijiazhuang 050037, China; 3 Baiyangdian Watershed Ecological Environmental Monitoring Center, Baoding 071051, China

**Keywords:** phthalate esters, Baiyang Lake, spatial distribution, source apportionment, risk assessment

## Abstract

As a kind of endocrine disruptor compounds, the presence of phthalate esters (PAEs) has become a global concern. In this study, the pollution levels and spatial distribution of sixteen PAEs were investigated. Their potential sources and eco-environmental health risk were discussed in Baiyang Lake and its upstream rivers during different periods. PAEs were detected in all of samples, ranging from 1215 to 3014 ng·L^−1^ in October 2020 and 1384 to 3399 ng·L^−1^ in May 2021. Dibutyl phthalate (DBP) and di-isobutyl phthalate (DIBP) were the predominant monomers, with a detection rate of 100% and the highest concentrations in the overlying water. Restricted by multiple factors, the spatial distribution difference between Baiyang Lake and its upstream rivers in October was more significant than in May. The source apportionment revealed that agricultural cultivation and disorderly use and disposal of plastic products were the primary factors for the contamination. The human health risk assessment indicated that eight PAE congeners did not pose significant carcinogenic and non-carcinogenic harms to males, females and children. However, the ecological risks of DBP, DIBP and di (2-ethylhexyl) phthalate to algae, crustaceans and fish species were moderate or high-risk levels. This study provides an appropriate dataset for the assessment of the pollution of PEs to the water ecosystem affected by anthropogenic activities.

## 1. Introduction

Phthalate esters (PAEs), as one of the most common plasticizers, are esterified from phthalic anhydrides and alcohol. The chemical structure of PAEs consists of a rigid plane aromatic ring and two adjacent nonlinear aliphatic side chains. They are a class of manufactured organic chemicals which are widely added in various substances, such as food packaging, personal care products, medical supplies, building materials, pesticides, and other commercial or industrial products [1,2]. As the European Union and the United States have successively issued decrees on PAEs production restrictions, China, India, Brazil and other developing countries have become the main global suppliers of PAEs [3]. According to data estimated by Plastics Europe Market Research Group, the global production of plastic in 2020 reached 367 million tons, and China was at the top of the list, with a contribution rate of 32%. Consequently, although PAEs have excellent performance in improving the flexibility and durability of plastic products, the environmental problems caused by PAEs have become increasingly serious because of their ubiquitous existence and tolerance to degradation [4]. PAEs are added to the polymer matrix by van der Waals force or hydrogen bond, rather than chemical bond. Therefore, in the whole process from production to waste, PAEs can be disintegrated, migrated and released into the environmental medium easily. In recent years, there is a growing literature about the study of PAEs in various environmental media, including soil (140–2130 μg·g^−1^) [5], water (27–4242 μg·L^−1^) [6], dust (9–808 μg·g^−1^) [7], and sediment (1–2 μg·g^−1^) [2].

In recent years, huge amounts of domestic and industrial wastewater have been discharged and the large-scale agricultural film have been applicated with the rapid development of urbanization, industrialization and agricultural modernization, aggravating the pollution of PAEs in the environment [8]. As semi-enclosed water bodies to store the upstream and regulate the downstream rivers, lakes showed higher PAE contamination levels, which is usually closely related to the pollution level of surrounding rivers [9]. Over the past decades, there have been reports on the level of PAEs pollution in the water environment of different regions in the world, such as lakes in Beijing in China [10], Taihu Lake in China [9,11], Asan Lake in Republic of Korea [12], Rhône River in France [13], and Tien Quang Lake in Vietnam [14]. The overall levels of PAE exposure were relatively high in China compared with those in other countries, and the most common PAEs detected in environment are DEP, DIBP, DBP and DEHP [8].

As an important kind of environmental endocrine disruptor compounds, PAEs can interfere with the functional balance of organisms and cause irreversible damage to the growth, development and reproduction of invertebrates, mammals, fish and amphibians [1]. Human beings can also continue to suffer potential damage from PAEs through diet, breathing, drinking water, and skin contact in daily life. PAEs may affect the human reproductive system, especially the motility of male sperm and increase the risk of female fertility [15]. In patients with long-term exposure to PAEs, renal and liver function will be impaired [16]. In view of the hazards of PAEs, the U.S. Environmental Protection Agency (EPA) has designated six congeners as priority pollutants for control. Considering the potentially negative impacts on human and aquatic organisms, more research has been conducted on human health and ecological risk assessment [17]. Many ecotoxicity tests have been carried out to provide appropriate amounts of data for risk assessment [18]. The risk quotient (RQ) is calculated according to the risk assessment Technical Guidance Document of the European Commission to assess the potential ecological risks of specific components [19].

Baiyang Lake, the largest shallow lake in the North China Plain, located in Xiongan New Area, which has started large-scale construction and has made fast progress in high-quality development as a new economic zone from 2017. It is essential for Baiyang Lake to maintain its ecosystem functions in this highly populated region. As most of shallow and macrophytic lakes, the ecological environment of Baiyang Lake is fragile and vulnerable to climate change and human activities. Due to the disorderly discharge of domestic wastewater and the destructive behavior of human beings, the water quality of Baiyang Lake has been seriously threatened since the 1980s and was once facing drying up [20]. In order to gradually restore the functions of the lake wetland, the Chinese government has adopted cross-basin ecological water replenishment and a number of environmental governance policies [21]. In recent years, research on various persistent toxic substances in Baiyang Lake has gradually been carried out, involving quinolones antibiotics, polycyclic aromatic hydrocarbons, perfluoroalkyl substances, fluorescent whitening agents, pharmaceuticals and personal products [20,22,23,24,25]. As a widely used plasticizer, PAEs were one of the important organic pollutants in the Baiyang Lake. However, there have been few comprehensive studies on their temporal variation and potential ecological and health risks are still unknown. The pollution characteristics and risk assessment of PAEs merit attention, particularly after local aquaculture and industrial activities were restricted. A better understanding of the occurrence and fate of PAE compounds within the water bodies is needed in order to provide a better risk assessment and to establish standards to control PAEs in the future.

In order to ensure the safety of water environment, the spatial distribution, source apportionment and risk assessment of PAEs were evaluated to improve our knowledge about the pollution distribution of PAEs, major sources and their risks. In accordance with the above, the purposes of this work were to: (1) investigate the occurrence and temporal-spatial distribution of PAEs in the overlying water; (2) trace and identify the potential sources of PAEs; and (3) evaluate the ecological and health risks from exposure to PAEs in the overlying water. As far as can be determined, this study was the first published account on the pollution characteristics and risk assessment of 16 PAEs in Baiyang Lake.

## 2. Materials and Methods

### 2.1. Chemical Reagents and Materials

Six PAEs (DMP, DEP, DBP, DEHP, DNOP and BBP) designated as priority pollutants by EPA and ten other prevalent PAE monomers, including di-iso-butyl phthalate (DIBP), di-methoxyethyl phthalate (DMEP), bis-(4-methyl-2-pentyl) phthalate (BMPP), bis-(2-ethoxyethyl) phthalate (DEEP), di-n-pentyl-phthalate(DPP), di-hexyl phthalate (DHXP), butoxyethyl phthalate (DBEP), di-cyclohexyl phthalate (DCHP), diphenyl phthalate (DPhP), and dinonyl phthalate (DNP), which were demonstrated to have negative effects on human beings and the eco-environment, were selected as target pollutants in this study. A mixed stock standard solution of 16 PAEs with the concentration of 1000 mg·L^−1^ were purchased from Alta-Scientific First Standard Inc. (Tianjin, China). The solid phase extraction (SPE) glass cartridge containing an CNWBOND HC-C18 filler was obtained from ANPEL Laboratory Technologies Inc. (Shanghai, China). High-performance liquid chromatography (HPLC) grade ethyl acetate, methanol, n-hexane, and methylene chloride were acquired from Kemiou Chemical Reagent Co., Ltd. (Tianjin, China). Neutral qualitative filter papers and 0.45 μm glass fiber filter membranes were supplied by Jinteng Experimental Equipment Co., Ltd. (Tianjin, China). To avoid sample pollution, the use of plastic containers was forbidden in this study. Before use, all glassware was soaked with dilute nitric acid for more than 24 h, rinsed separately with distilled water and ultra-pure water at least five times, and then dried at 450 °C for 6 h to deduct background pollution.

### 2.2. Study Area and Sample Collection

Baiyang Lake is characterized by the great influence of human activities. Thirty-nine water villages, with a population of 100,000, are embedded in the lake, and 89 other villages are distributed around the lake [22]. As a typical northern wetland, Baiyang Lake has an important ecological function. Due to the perennial drought and lack water resource in the north of China, most of the nine upstream rivers are in danger of drying up, apart from Fu River and Xiaoyi River, which receive the tailwater from the upstream urban sewage treatment plant. Almost no natural overlying water flows into the lake.

**Figure 1:**
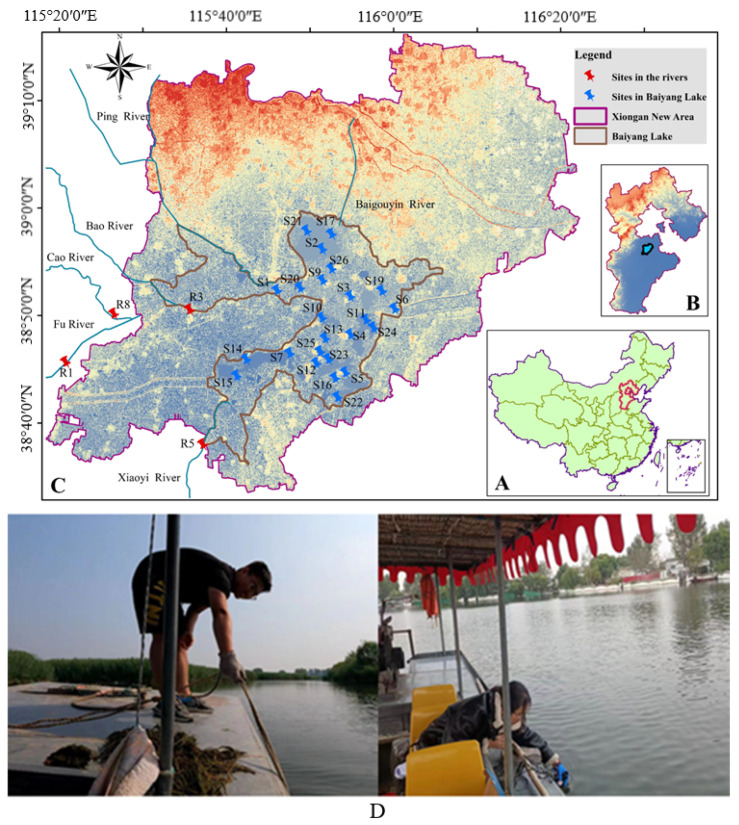
Locations of Hebei Province (**A**), Xiongan New Area (**B**), and sampling sites in Baiyang Lake and its upstream rivers (**C**), and the sampling was conducted in the field (**D**).

In order to comprehensively investigate the pollution level of the target PAEs in Baiyang Lake, 28 sampling sites were set up in Baiyang Lake and its upstream rivers, including Fu River, Xiaoyi River and Bao River, which were watery in October. The detailed location information of the samples is shown in Figure 1. Among the 28 sampling sites, four sites (R1, R3, R5 and R8) were located in the upstream rivers, and 24 of them were located in the Lake. Therein, 11 sites (S1, S3 to S7, S9, S14, S20, S22 and S26) were located near the water villages in Baiyang Lake and were strongly affected by human activities, and 13 sites (S2, S10 to S13, S15 to S17, S19, S21, S23 to S25) were located in natural areas with less anthropogenic activity. Detailed sampling information is given in the Supplementary Information (Table S1).

Twenty-eight overlying water samples were collected at the selected sampling sites in October 2020, whereas 27 water samples were collected in May 2021, except for Site R1 due to certain restrictive conditions. The overlying water samples were collected at 0.5 m below the water surface using a Plexiglass water sampler. Prior to each sampling, the sampler was rinsed several times to avoid possible cross-contamination. All samples were sealed in 1.5 L amber glass bottles and transported back to the laboratory within 12 h. Each sample was filtered through a 0.45 μm glass fiber filter (Whatman, UK) using a filtration device consisting of a vacuum pump to remove suspended solids and bacterial residues. Then, the samples were refrigerated at 4 °C before further chemical analysis. The determination of all samples was completed within 7 days.

The basic properties of water, including pH, dissolved oxygen (DO) and temperature, were measured in situ using a portable water quality parameter tester (Waltham, MA, USA). The main physico-chemical indexes, including chemical oxygen demand (COD), total nitrogen (TN), total phosphorus (TP), total organic carbon (TOC), permanganate index (COD_Mn_), ammonia nitrogen (NH_4_^+^-N), nitrite nitrogen (NO_2_^−^-N) and nitrate nitrogen (NO_3_^−^-N), were determined by ultraviolet visible spectrophotometry.

### 2.3. Sample Pretreatment and Instrumental Analysis for PAEs

The filtrated water was extracted by the SPE system, based on the method of previous literature, with slight modifications [26]. In brief, a 1.0 L aliquot of each filtered sample was taken out and several drops of c oncentrated sulfuric acid were added into the water samples to adjust the pH to 7.5 mL; methanol was added and shaken evenly. SPE column was cleaned twice with 5 mL of n-hexane, activated successively by 6 mL of methanol, and finally washed using 6 mL of ultrapure water before use. Each solvent retained 5 to 10 min to fully infiltrate the packing and passed through the extraction column slowly at the flow rate of 1 mL·min^−1^. During the wash with the ultrapure water, the liquid level remained tangent to the filler to keep the filler wet. The water sample was uniformly passed through the SPE column at a flow rate of 5 mL·min^−1^ to achieve the enrichment effect.

After washing the column with 6 mL ultra-pure water, 8 mL of dichloromethane and ethyl acetate (*v*/*v* = 1/1) was added to elute the target analytes at a flow rate of 1 mL·min^−1^. The eluent was dehydrated and dried by anhydrous sodium sulfate and then evaporated to near dryness under high-purity nitrogen flow at 38 °C. Eventually, the residue was redissolved with n-hexane to 1 mL for quantitative analysis.

An Agilent 7890B gas chromatography, combined with an Agilent 5977A mass spectrometer (GC-MS) (Palo Alto, CA, USA), was used for separation and determination of the target PAEs in electron impact mode, equipped with a DB-5MS capillary column (30 m × 0.25 mm × 0.25 μm) (Folsom, CA, USA). The stepped temperature program was initiated at 60 °C for 2 min, increased to 220 °C at a rate of 20 °C·min^−1^ and held for 2 min, 5 °C·min^−1^ up to 250 °C and held for 1 min. Finally, the temperature was increased to 290 °C at 20 °C·min^−1^, which was maintained 10 min. 1.0 μL analyte was injected into GC-MS without shunt in a constant current splitless injection mode, and the selected ion monitoring mode was used for determination.

### 2.4. Quality Control and Quality Assurance

In this study, 28 overlying water samples were collected in October 2020, and 27 water samples were collected in May 2021. To monitor the potential contamination during the sampling campaign and laboratory background, two to three parallel samples with procedural blanks were processed for each quarterly sampling site, and every batch of ten samples were processed with field blanks, procedural blanks and solvent blanks. The eluent and elution times were optimized during sample pretreatment (Figure S1). The method recoveries for water of the studied PAEs ranged from 84.91% to 114.0%. The correlation coefficients (r) of all the PAEs calibration curves were higher than 0.99. The method detection limits (MDLs) were 0.002 to 0.064 μg·L^−1^ in the water, and the method quantification limit (MQL) was assigned as a value of the MDL multiplied by 3 (MQL = MDL*3). Please see Table S2 for details regarding each compound.

### 2.5. Data Analysis

The statistical analysis, including principal component analysis (PCA) and Pearson correlation analysis, were conducted using SPSS 26.0 software (SPSS Inc., Chicago, IL, USA). The data analysis and visualization were performed using Origin Pro 2021 software (OriginLab Corporation, Northampton, MA, USA) and ArcGIS 10.7 (Esri Corporation, Redlands, CA, USA).

### 2.6. Risk Assessment

#### 2.6.1. Human Health Risk Assessment

Health risk assessment mainly evaluates the effects or harm caused by toxic chemicals according to the different approaches and ways of human exposure. The main exposure routes of PAEs to the local residents in Baiyang Lake are direct oral intake by drinking water and daily skin exposure. Their corresponding average daily exposure doses *ADD_dri_* and *ADD_der_* were calculated as expressed:

(1) $${\;\mathrm{ADD}}_{\mathrm{dri}}=\frac{{\mathrm{CC}\;\times \;\mathrm{RM}\;\times \;\mathrm{MEC}\;\times \;\mathrm{EF}}_{\mathrm{dri}}{\;\times \;\mathrm{ED}\;\times \;\mathrm{IR}}_{W}}{\mathrm{BW}\;\times \;\mathrm{AT}}$$

(2) $${\;\mathrm{ADD}}_{\mathrm{der}}=\frac{0{.5\;\times \;\mathrm{CC}\;\times \;\mathrm{RM}\;\times \;\mathrm{MEC}\;\times \;\mathrm{PC}\;\times \;\mathrm{SA}\;\times \;\mathrm{EF}}_{\mathrm{der}}\;\times \;\mathrm{FE}\;\times \;\mathrm{ED}\;\times \;\frac{6\tau \;\times \;\mathrm{TE}}{\pi }}{500\;\times \;\mathrm{BW}\;\times \;\mathrm{AT}\;\times \;F}$$

where *CC* is conversion coefficient; *RM* is boiling residue ratio; *MEC* is the pollutant concentration in water (μg/L); *IR_W_* is the daily drinking water volume; *F* is the intestinal adsorption ratio; *BW* is the body weight; *EF* is the exposure frequency; *ED* is the exposure duration; *SA* is the surface area of the skin; *FE* is the bathing frequency; *TE* is bath time; *AT* is the average exposure time; and *PC* is the skin permeability constant of chemical substance. The specific values of each parameter are shown in Table S3.

The noncarcinogenic and carcinogenic risks by the exposure of PAEs were estimated according to the methods recommended by the USEPA exposure assessment guidelines [27]. *ILCR* and *HI* represent carcinogenic risk and non-carcinogenic risk, respectively, and their calculation formulas are:

(3) $${\;\mathrm{ILCR}=\mathrm{ADD}}_{\mathrm{dri}}{\;\times \;\mathrm{SF}}_{\mathrm{dri}}{+\mathrm{ADD}}_{\mathrm{der}}{\;\times \;\mathrm{SF}}_{\mathrm{der}}$$

(4) $$\;\mathrm{HI}=\frac{\;{\mathrm{ADD}}_{\mathrm{dri}}}{{\mathrm{RfD}}_{\mathrm{dri}}}+\frac{{\mathrm{ADD}}_{\mathrm{der}}}{{\mathrm{RfD}}_{\mathrm{der}}}$$

where *ADD_dri_* and *ADD_der_* are daily exposure dose through drinking water and skin contact [mg·(kg·d)^−1^]; *RfD_dri_* and *RfD_der_* are long-term intake reference dose of drinking water and skin contact route [mg·(kg·d)^−1^]; and *SF_dri_* and *SF_der_* are the carcinogenic slope factors through drinking water and skin contact [(kg·d)·mg^−1^], respectively. All values are derived from the relevant parameters of comprehensive risk information released by the USEPA and are also shown in Table S3.

According to the USEPA guidelines [28], one in a million chance of human cancer risk (ILCR = 10^−6^) is considered an acceptable security threshold. The different health risk levels were established as follows: negligible risk to organisms for ILCR values lower than 10^−6^; medium risk for values between 10^−6^ and 10^−4^; and high risk for values higher than 10^−4^.

Due to the lack of some reference data, the non-carcinogenic risk for only eight types of PAEs, including DMP, DEP, DIBP, DBP, DHXP, BBP, DEHP and DNOP, were assessed. HI is hazard index. If the value of HI is greater than 1, humans are considered to be exposed to non-cancer risks.

#### 2.6.2. Ecological Risk Assessment

Risk quotient (*RQ*) method was used to perform the potential ecological risk of PAEs to fish, algae, and crustaceans in Baiyang Lake. The HQ for PAEs was calculated using the exposure concentration (*MEC*) and the predicted no-effect concentrations (*PNEC*). The *RQ* was calculated as follows:

(5) $$\mathrm{RQ}=\frac{\mathrm{MEC}}{\mathrm{PNEC}}\;$$

where *MEC* (μg·L^−1^) is the PAEs concentration in overlying water for each sampling site (Table S4). As a safe threshold for compound concentration [18], the *PNEC* (μg·L^−1^) was calculated as follows:

(6) $$\mathrm{PNEC}=\frac{{L(E)C}_{50}}{\mathrm{AF}}\;$$

where *L(E)C_50_* represents the half lethal effect concentration (μg·L^−1^) of the tested species and *AF* represents an assessment factor. The specific *AF* was defined as 100 with one available long-term NOEC; as 50 with two available long-term NOECs; and as 10 with three available long-term NOECs for the three different trophic levels’ chronic toxicity data. If only acute toxicity data were screened, AF was defined as 1000. Toxicological data are mainly obtained from the ECOTOX of USEPA and several published literature reports [29]. Detailed data on aquatic organisms at different trophic levels are shown in Table S5.

The HQ value was classified into three levels to assess the ecological risk, as below [30]:

HQ < 0.01: no ecological risk;

0.1 ≤ HQ < 1: low ecotoxicological risk;

HQ ≥ 1.0: high ecological risk.

## 3. Results and Discussion

### 3.1. Occurrence and Composition Profiles of PAEs in the Baiyang Lake

A total of 55 overlying water samples were detected in this study. Among the 16 target PAEs, 12 PAEs were detected, except for DMEP, DPP, BMPP and DNP. Meanwhile, 11 PAEs were detected, except for DMEP, DPP, BMPP, DNP and DPhP, in May. The individual concentrations of the detected 12 PAEs are summarized in Table 1. The occurrence levels of the detected PAEs in October in overlying water samples ranged from 1215 ng·L^−1^ to 3014 ng·L^−1^, with an average value of 1840 ng·L^−1^ (*n* = 28). However, the total concentrations of the 11 target PAEs in May was in the range of 1384 to 3399 ng·L^−1^, with an average concentration of 2176 ng·L^−1^ (*n* = 27). DBP and DIBP were the predominant monomers with the highest concentrations in the water of Baiyang Lake.

Furthermore, the results also indicated that DMP, DEP, DIBP, DBP, DBEP, DCHP, and DEHP could be detected in all samples. The detection rates of DEEP, DNOP, BBP, DPhP, and DHXP in October were 67.86%, 60.71%, 53.57%, 50.00%, and 42.86%, respectively. However, DNOP, BBP, DHXP, and DEEP were separately detectable at rates of 81.48%, 66.67%, 62.96%, and 55.56% in May (Figure S2).

According to the average values of the individual PAEs, DBP (354.7 ng·L^−1^), DIBP (330.1 ng·L^−1^), DCHP (234.2 ng·L^−1^), DBEP (226.9 ng·L^−1^) and DEHP (189.1 ng·L^−1^) were the preponderant PAE species, with a total concentration contribution of 72.57% in October. DBP (831.1 ng·L^−1^), DIBP (325.3 ng·L^−1^), DBEP (374.8 ng·L^−1^), DCHP (125.9 ng·L^−1^) and DMP (113.8 ng·L^−1^) were the preponderant PAE species, with a total concentration contribution of 81.37% in May. As far as the contribution of each component to ∑PAEs is concerned, a few species, including DBEP, DIBP and DCHP, which are not incorporated in the priority-control list, occurred with high fractions. Liu [29] also obtained similar investigation results regarding distribution of PAEs in the surface sediment-pore water system of the Haihe River. Compared with October, the average detected concentration of DBP, DBEP and DMP in May increased by 476.4 ng·L^−1^, 147.9 ng·L^−1^, and 28.47 ng·L^−1^, respectively. In contrast, the average concentrations of DCHP, DEHP, DEP, and DNOP showed a downward trend, with the reduction values of 108.4 ng·L^−1^, 103.1 ng·L^−1^, 45.27 ng·L^−1^, and 41.85 ng·L^−1^, respectively. BBP, DEEP and DHXP concentrations did not change significantly. The average level of DBP and DEHP in this study (354.7 ng·L^−1^ and 189.1 ng·L^−1^ in October; 831.1 ng·L^−1^ and 85.98 ng·L^−1^ in May) did not exceed the stipulated level (DBP: 3 μg·L^−1^; DEHP: 8 μg·L^−1^) according to the environmental quality standards of China for overlying water. Nevertheless, the rapid growth of DBP demonstrated that great attention should be paid to it.

In brief, DBP and DIBP were the dominant congeners of PAEs in Baiyang Lake in terms of composition and concentration. This result is slightly different from previous studies, which reported DBP and DEHP were the main components of PAEs contamination [14,31]. This difference was mainly due to the fact that only the six PAEs were measured, including DMP, DEP, DBP, BBP, DEHP and DNOP, which are listed as priority pollutants and endocrine-disrupting chemicals by the USEPA [32]. In fact, DIBP has been widely detected in the environment as a substitute and homologue of DBP.

Over the last decade, several studies have systematically reported and compared the PAEs pollution levels of many lakes in China; it cannot be ignored that existing literature on the pollution level of Baiyang Lake is scarce. In this study, 17 studies on the concentrations PAEs in aquatic environmental media at home and abroad, published from 2008 to 2021, were summarized in Table 2, in order to comprehensively evaluate the pollution levels in Baiyang Lake.

The results show that the ∑_16_PAEs in the overlying water of the Baiyang Lake is similar to that of the Lake of Guang Zhou, China [33], Changjiang River Estuary, China [34] and Pearl River, China [26], and higher than Rhône River, France [13], Asan Lake, Republic of Korea [12], Kaveri River, India [35] and Lakes of Beijing, China [10], while significantly lower than that from economically and industrially developed Songhua River [36], Chaohu Lake, China [31], Jiulong River, China [37], Taihu Lake, China [9], Tien Quang Lake, Vietnam [14], Bohai Sea, China [17], and Hangzhou Bay, China [8].

**Table 2:** Comparison of the PAE concentrations with other areas in the world.

Survey Region	Number and Range of ∑PAEs (μg·L^−1^)	Reference
Lake of Guang Zhou, China	∑_15_PAEs: 1.69–4.72	[33]
Chaohu Lake, China	∑_6_PAEs: 1.21–17.95	[31]
Lakes of Beijing, China	∑_15_PAEs: 0.39–3.18	[10]
Songhua River, China	∑_6_PAEs: 6.95–44.72	[36]
Kaveri River, India	∑_2_PAEs: 0.31–1.64	[35]
Jiulong River, China	∑_6_PAEs: 3.48–17.70	[37]
Taihu Lake, China	∑_6_PAEs: 0.72–13.00	[9]
Asan Lake, Republic of Korea	∑_14_PAEs: ND-2.29	[12]
Pearl River, China	∑_14_PAEs: 0.01–6.72	[26]
Changjiang River Estuary, China	∑_16_PAEs: 0.18–3.42	[34]
Tien Quang Lake, Vietnam	∑_10_PAEs: 19.60–127.00	[14]
Rhône River, France	∑_7_PAEs: 0.10–0.54	[13]
Hangzhou Bay, China	∑_10_PAEs: 7.31–22.90	[8]
Bohai Sea, China	∑_16_PAEs: 0.01–52.58	[17]
Baiyang Lake, China	∑_16_PAEs: 1.21–3.39	This study

### 3.2. Spatial and Temporal Distribution and Variation of PAEs

**Figure 2:**
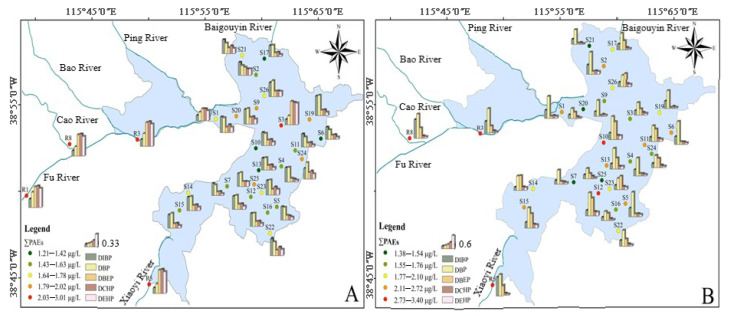
The concentrations of five preponderant PAE species and total concentrations detected in each sampling site in October (**A**) and May (**B**).

The spatiotemporal distributions of the total PAEs and the five preponderant PAE species at each sampling site in October and May are shown in Figure 2A,B, respectively. The total concentrations of 18 sites in May were higher than in October, accounting for 66.67% of the total sites, which may be attributed to increased agricultural chemical application and discharge from sewer systems due to high hydraulic loads and associated lower treatment efficiencies, as well as seasonal increases in atmospheric deposition and catchment runoff [19], causing more serious pollution to some sites. During October, DBEP, DEHP and DCHP contributed more to the total concentration of the upstream rivers, while the contribution rates of DIBP and DBP to the total concentration in the lake region were lesser, although the dilution effect of water is more prominent due to the increase of water volume and flow rate [33]. The contribution rate of DBEP, DCHP and DEHP to total concentrations in October was significantly higher than that in May, but there was no obvious difference in pollution components between rivers and the lake in May (Figure 2). DBP and DIBP were still the dominant pollutant congeners of PAEs.

Spatially, the maximum total concentration of overlying water was found in R3 (3.014 μg·L^−1^) in October and in R8 (3.399 μg·L^−1^) in May, respectively. The total concentrations in the Fu River, Xiaoyi River and Bao River were higher than in Baiyang Lake, indicating that the water environment of the upstream rivers still exerted great pressure on the lake area. The main source of water supply of the Fu River upstream is the industrial and agricultural wastewater and domestic sewage of Baoding City in the upper reaches, especially the tail water of the sewage treatment plants. Since specific PAEs usually serve as plasticizers in plastic products, such as polyvinylchloride (PVC) and polyvinyl acetates, large consumption amounts of PAEs in urban areas would increase the quantity of PAEs discharged into the river [29]. A large number of existing sewage treatment plants are mostly designed for nitrogen and phosphorus removal, while ignoring the impact of drug residues, personal care products, food additives and endocrine disruptors on water quality safety [38]. The upper reaches of Xiaoyi River are typical and famous industrial zones in the Hebei Province, where a number of printing enterprises are gathered. The treated printing and dyeing wastewater still contain a large number of microfibers adsorbed by dyes, which are discharged into overlying water [39].

The coefficient of variation of the detected PAEs monomers in October and May overlying water samples ranged from 1.22 to 74.59% and 22.98 to 74.11%, respectively (Table 1). These indicate that the spatial distribution of main components of PAEs in October showed obvious differences, while the coefficient of variation of total PAEs monomers increased significantly, suggesting that the spatial distribution difference of sites increased further in May. The concentrations of DBEP, DEHP and DCHP in the upstream rivers account for a large proportion, while DIBP and DBP have a higher proportion to the total concentration in the lake area. As the confluence of Fu River and Baiyang Lake, similar pollution characteristics occurred between S1, R1 and R3. Different from ordinary lakes, Baiyang Lake has unique hydrographic and geomorphic features, which is specifically explained by the interwoven composition of lakes of different sizes and villages. Therefore, the sites located in the rural area near villages (S3–S7, S9, S20, S26) were more obviously affected by human activities, and the degree of pollution was more serious than that in natural areas (S2, S11, S17, S21, and S24). Although S25 is located in a natural site, it is surrounded by the villages of Dongtianzhuang, Datianzhuang and Beitianzhuang. Concurrently, the relatively closed waterbody is also a major factor of pollution accumulation.

### 3.3. Sources Apportionment by Pearson Correlation Analysis and Principal Component Analysis

In the past decades, Baiyang Lake has confronted the process of severe eutrophication, resulting in the accumulation of a large amount of nutrients in the sediment. So far, this situation has not been fully improved, and endogenous pollution still poses a serious threat to the water environment of Baiyang Lake. The measured values of several water quality indexes in different seasons were shown in Table S6. Analysis of the correlations between conventional index and PAEs in overlying water have been conducted (Figure S3). In October, nitrogen index (TN, NH_4_^+^, and NO_3_^−^) had positive correlation with DBEP, DCHP and DEHP, but negative correlation with DIBP. In October and November, the dominant aquatic plants, such as cattail and bulrush, entered the recession period. If not harvested, the residue will decompose and cause secondary pollution, resulting in an increase in nitrogen. Like other organic compounds, PAEs are also utilized as a carbon source by microorganisms [16]. The high molecular weight phthalates, such as DEHP and DCHP, are less degradable, while low molecular weight phthalates, like DEP and DBP, can degrade easily. In May, DEP, DBP, DIBP, DEEP and conventional indexes (TN, TP, NH_4_^+^, and NO_3_^−^) showed significant positive correlation; other indexes were not significantly correlated with the concentration of PAEs. This may be due to the rise of temperature, the growth of plants, the increase of dissolved oxygen, the acceleration of water flow and frequent human activities providing conditions for the reproduction, growth and diversity of aerobic denitrifying bacteria [40]. The rapid consumption of nutrients, such as nitrogen and phosphorus, will cause the PAEs of short molecular chains to be decomposed.

**Figure 3:**
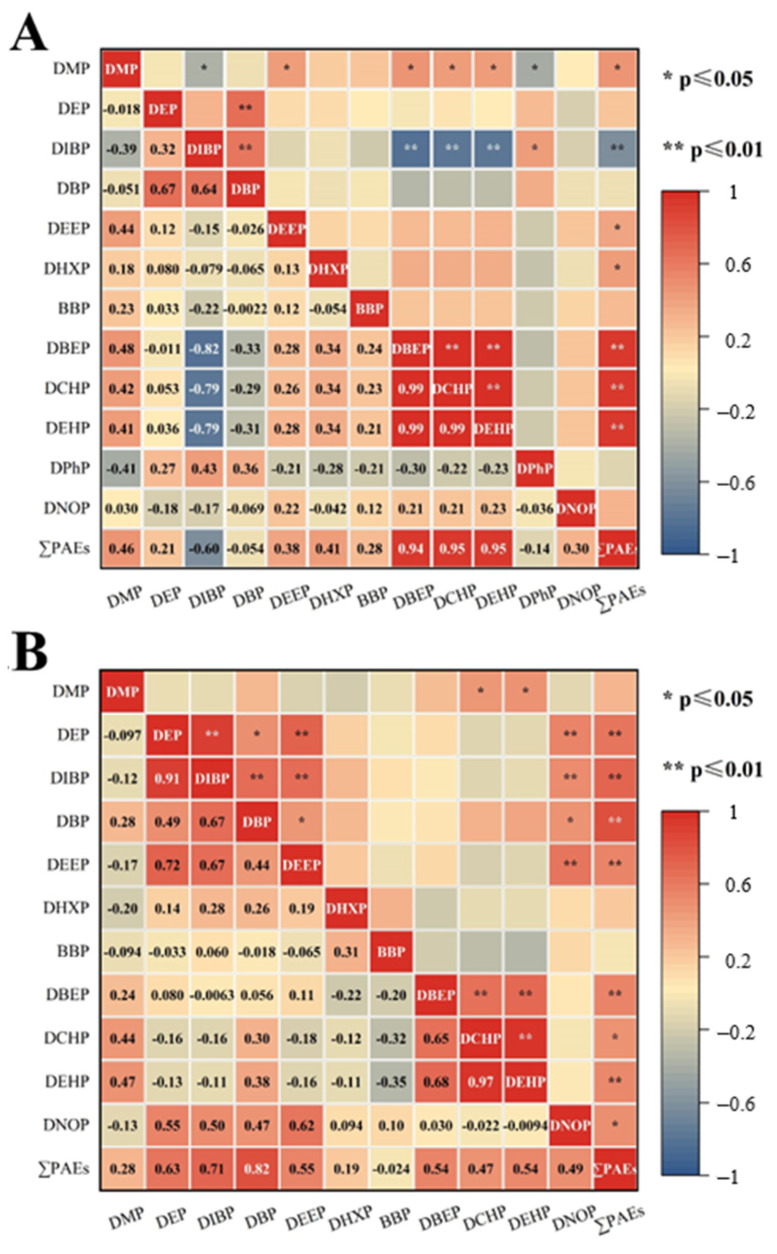
Pearson correlation analysis between individual PAE and ∑PAEs in October (**A**) and May (**B**). **: the correlation was significant at *p* ≤ 0.01 level. *: the correlation was significant at *p* ≤ 0.05 level.

Furthermore, PCA was conducted to perform factor analysis on the PAEs in the overlying water of Baiyang Lake, as shown in Table S7. According to the principle that the eigenvalue is greater than 1, we extracted four principal components to explain 74.87% of the variance in October and three principal components to explain 71.68% of the variance in May (Table S7). In October, PC1 explained 38.94% of the total variance, with loading of −0.78, 0.96, 0.98, and 0.98 for DIBP, DBEP, DCHP and DEHP, respectively. The results were in agreement with the Pearson correlation analysis in Figure 3, which inferred a significant correlation between DIBP, DBEP, DCHP, DEHP and ∑PAEs, indicating these four homologues are the main components of October. PC2 accounted for 15.51% of the total PAEs in these samples and was dominated by low molecular PAEs with short chain for DEP (0.894) and DBP (0.862). PC3 contained DMP, DEEP and DPhP, while PC4 used the remaining PAEs to explain 9.85% of variance. The main use of PAEs as plasticizer is widely used in PVC, PC, PS, PET, ABS and other plastic-related products to improve its technical properties, and about 80% of PAEs is used for this purpose. In China, PAEs are extensively applied in daily necessities, food packaging, medical equipment, industrial piping, upholstery, agriculture plastic films, etc. [41]. DBEP, DCHP and DEHP were significantly correlated (r > 0.9, *p* < 0.01). It is generally believed that monomer substances with significant correlation may have homology [7]. DCHP is also a branching alkyl chain PAE congener with similar properties to DEHP [4]. DEHP is still the most widely used plasticizer in the world because of its excellent properties and low price. As a plasticizer, DCHP can make the plastic surface shrink closely so as to prevent moisture and volatilization, so that the surface of plastic products become smooth and improve the tactile sensation. DBEP is also a common plasticizer used to prevent the build-up of a static charge on a plastic surface. In contrast, due to the small number of carbon atoms in the side chain, DBP, DMP and DEP are widely used as non-plasticizers in cosmetics, PVC coatings, nail polish and other personal care products. In conclusion, PC1 and PC4 were mainly related to plasticizers in various sources; PC2 and PC4 were mainly associated with the use of non-plasticizers like cosmetics, PVC coatings, and personal care products.

In May, it can be seen from the composition matrix that PC1 mainly contains DEP, DIBP, DBP, DEEP and DNOP, while DMP, DBEP, DCHP, and DEHP belong to PC2; PC3 mainly includes DHXP and BBP. As in October, the results of principal component analysis and correlation analysis can verify each other. DEP (r = 0.628), DIBP (r = 0.706), DBP (r = 0.823), DEEP (r = 0.555), DBEP (r = 0.544), DCHP (r = 0.469), DEHP (r = 0.535) were significantly correlated with ∑PAEs (*p* < 0.01) in Table S7. DBP and its substitute DIBP are also widely used. Because of low molecular weights, DBP and DIBP can be used as pesticides, air fresheners and other personal care products. DBP is also used in special adhesive formulations, cellulose esters, and epoxy resins [7]. In addition, the results of the investigation of PAEs from 46 lakes in China show that the concentration of DIBP is affected by many agricultural factors, such as plastic film mulching area, the proportion of cultivated land, and the application of chemical fertilizers and pesticides [42]. With agricultural modernization, plastic mulching and plastic film greenhouses have become very popular for agricultural production all over China. As demonstrated, DIBP and DBP have been detected in farmland soils in several provinces [5]. On the other hand, as a kind of common high-risk pesticide auxiliary, PAEs uses its biological and chemical activities to improve the physical and chemical properties of pesticide preparations and improve the convenience and efficiency of pesticide application. The extensive use of pesticides and fertilizers in the season of agricultural planting in May will cause agricultural non-point source pollution. Pesticides, chemical fertilizers, agricultural films and other substances containing PAEs, which are widely used in agricultural activities, will continue to be enriched in soil particles if they are not degraded in time. They enter the water system through surface runoff, rainwater erosion, groundwater erosion and other ways, causing pollution to the aquatic environment [43]. Therefore, it is particularly necessary to strengthen the supervision of PAEs-related products in agricultural activities.

The dry and wet deposition of the atmosphere also plays an important role in the change of PAEs concentration in water. Residents around Baiyang Lake have long taken plastic manufacturing as their main economic industry, producing a large number of plastic products. Located in the upper reaches of the Baigouyin River is a famous luggage production base in China, which uses lots of light industrial products, such as artificial leather and synthetic rubber, in its production process. The research point of view shows that the volatilization strength coefficient of PAEs in artificial leather is much higher than that of typical plastic packaging materials, such as PVC wallpaper, plastic bags and plastic toy products. Simultaneously, packaging, building and construction represent the largest end-use markets for plastic products by far. The degradation rate and half-life of PAEs decrease with the increase of side chain alkyl length and n-octanol-water partition coefficient. Some PAEs monomers, such as DEHP and DIBP, are hydrolyzed, photolyzed and biodegraded slowly in freshwater lakes and accumulated in the water for a long time. In short, long-distance atmospheric transport will also affect the fluctuation of pollution in Baiyang Lake.

### 3.4. Risk Assessment of PAEs in the Baiyang Lake

#### 3.4.1. Human Health Risk Assessment

**Figure 4:**
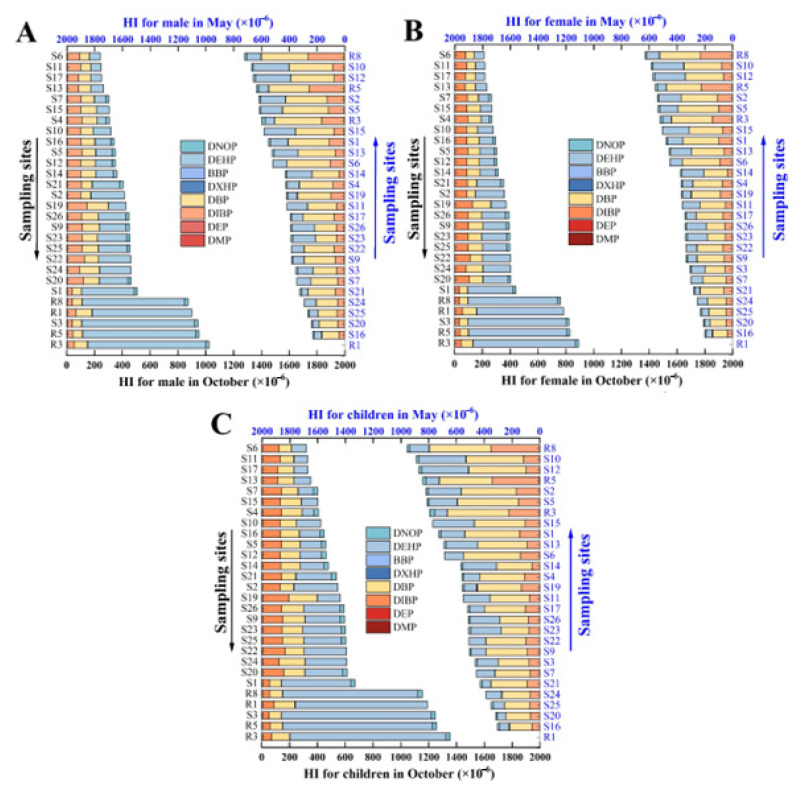
Results of human health risk assessment via drinking water and skin contact for males (**A**), females (**B**) and children (**C**) in October and May.

When calculating the human health risks of different compounds, it is usually necessary to consider the synergistic effects among them—that is, the risk values of different exposure routes are calculated [44]. According to the relevant parameters and formulas listed, the average concentration was used to evaluate the health risk of eight kinds of PAEs monomers in males, females and children, respectively. The assessment results were shown in Figure 4.

For the non-carcinogenic risk of PAEs to adults and children through both direct drinking and skin contact, all the HI values were not greater than 1, indicating that the non-carcinogenic risk to both adults and children was within the safe threshold regardless. Specifically, the orders of magnitude of HI for different populations via direct drinking varied in a range of 2.09 × 10^−9^ to 3.53 × 10^−6^, while those through direct skin contact ranged from 4.98 × 10^−12^ to 8.85 × 10^−9^, about 10^3^ to 10^5^ times lower than that of HI through direct drinking water. Hence, direct drinking was a possible exposure pathway for PAEs in Baiyang Lake impacting on human health. According to previous studies, food, tap water and indoor air are the main sources of human PAEs intake, and the contribution rate of tap water is second only to food [1]. Simultaneously, the results of direct drinking route showed that the risk of the eight monomers of 8 PAEs to different populations was children > male > female. In other words, children were more susceptible to PAE damage than the adults. This result agrees with previous studies and can be mutually corroborated [45]. It may be due to the fact that toddlers have more hand-to-mouth contact activities and the differences in selected parameters. Studies have indicated that chronic exposure to PAEs can disrupt endocrine activity, hamper reproduction, and affect normal development [45]. The risk of skin contact pathway for females is higher than males and children, possibly due to differences in bathing duration. Although skin contact poses a small threat to health, it does not mean that it can be ignored. Therefore, it is particularly necessary to objectively and fairly evaluate the effects of different ways of skin contact on human health.

The HI values of individual compounds from the highest to the lowest were: DEHP > DBP > DIBP > DNOP > BBP > DHXP > DEP > DMP. DEHP, DBP and DIBP and its homologues have the highest numerical contribution to the non-carcinogenic risk of ∑PAEs. As the only carcinogenic risk, meanwhile, DEHP values for different populations range from 2.96 × 10^−10^ to 9.88 × 10^−10^. DEHP is recognized as a class 2B carcinogen by the International Cancer Society and is a potential cause of breast cancer in women and prostate cancer in men [46]. Although there is a relative lack of carcinogenic assessment data on DNOP, there are still data showing that most of the existing sewage treatment processes have a very low removal rate of DNOP, thus posing an immeasurable threat to the environment. Fortunately, the carcinogenic risk and non-carcinogenic risk of the eight kinds of PAEs in Baiyang Lake water environment are all within the safety threshold. However, we still need to strengthen the monitoring of DEHP, DNOP, DBP and DIBP, synchronously, and choose a more appropriate and reliable exposure pathway for assessment.

#### 3.4.2. Ecological Risk Assessment

**Figure 5:**
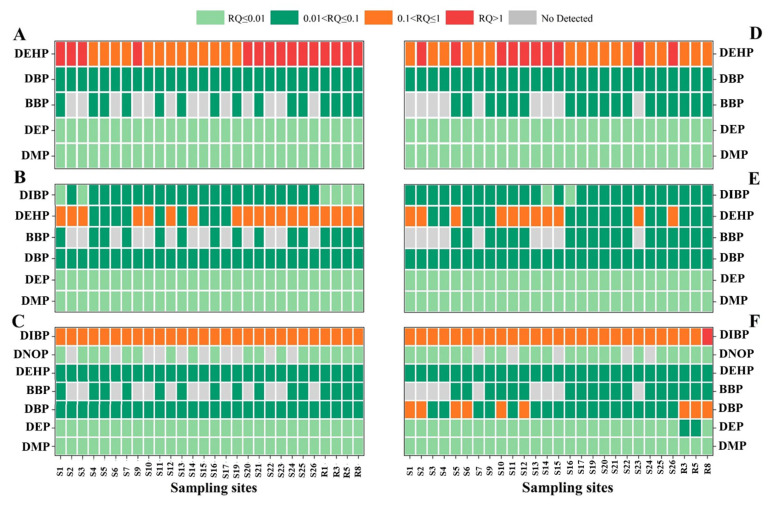
Results of ecological risk assessment of the selected PAE species exposed to the sensitive aquatic organisms: algae (**A**), crustacean (**B**), fish (**C**) in October and algae (**D**), crustacean (**E**), fish (**F**) in May.

The toxicological data for the priority PAEs cover a wide range of taxa and species; however, information is extremely limited for the other congeners recently developed for new applications or as substitutes for priority PAEs [11]. In this study, six priority PAEs and DIBP were selected to carry out ecological risk assessment for algae, crustacean and fish species. The assessment results are shown in Figure 5. The RQ values of the studied PAEs for algae followed the sequence of DEHP > DBP > BBP > DMP > DEP. The RQ values for crustaceans followed the order of DEHP > DBP > BBP > DIBP > DEP > DMP, and the RQ values for fish followed as DIBP > DBP > DEHP > BBP > DEP > DMP. These results are consistent with the results of the Taihu Lake Basin [11] and Jiulong River in China [37].

For individual species, the RQs of DEHP to algae was high, at 53.57% of total sampling sites in October and 37.04% in May, with the rest being moderate risk. Similarly, the RQs of DEHP to crustaceans was moderate, at 67.86% of total sampling sites in October and 40.74% in May. The rest was low-risk. In addition, DMP, DEP, DIBP, DBP and BBP posed low- or no risk to both algae and crustaceans. Nevertheless, most of the RQs of DIBP to fish were moderate risk in different months. The RQs of DBP to fish was moderate at 33.33% of total sampling sites in May, while the rest was low risk. In general, the ecological risk of upstream rivers is higher than that in Baiyang Lake. The sites with higher ecological risk are more commonly located in the rural areas near villages in Baiyang Lake, which is greatly affected by human activities. As the typical PAEs pollutants in Baiyang Lake, the ecological risk of DBP with its homolog DIBP and DEHP needs to be paid special attention. Toxicity information for DBEP, DEEP, DPhP and others is deficient in the previous literature, which makes the risk assessment become impossible in this study.

## 4. Conclusions

This study provided the first comprehensive overview of the pollution levels, distribution, sources and risks of PAEs in the waters of Baiyang Lake and its upstream revers. PAEs were present in all samples, with total concentrations from 1215 ng·L^−1^ to 3399 ng·L^−1^, which indicated that PAEs are already ubiquitous in the studied area. DBP and DIBP were the dominant compounds in both October and May. It was disclosed that the potential sources of PAEs were the anthropogenic activities closely linked to the concentration in Baiyang Lake. The results of human health risk assessment confirmed that eight PAEs did not cause salient health harm to males, females and children. The ecological risk assessment suggested that DBP, DIBP and DEHP harm to algae, crustaceans and fish appeared to be moderate or high risk in succession. This work could help to identify the PAE potential contamination levels, sources and risks, and provides useful support for the future environmental management and remediation efforts.

## Figures and Tables

**Table 1:** Statistical summary of the detected PAE concentrations in the overlying water in Baiyang Lake.

PAEs	October (N = 28)					May (N = 27)				
	Concentration (ng·L^−1^)			CV (%)	Detection Rate (%)	Concentration (ng·L^−1^)			CV (%)	Detection Rate (%)
	Min	Max	Average			Min	Max	Average		
DMP	79.91	90.61	85.29	3.31	100	67.74	247.6	113.8	35.96	100
DEP	78.71	84.37	80.47	1.39	100	22.21	83.73	35.20	33.65	100
DIBP	137.5	511.4	330.14	29.42	100	145.4	921.8	325.3	58.41	100
DBP	226.4	536.2	354.7	21.38	100	42.63	1196	831.1	28.98	100
DEEP	ND	72.62	69.91	1.22	67.86	ND	216.2	62.55	74.11	55.56
DHXP	ND	61.63	58.82	1.57	42.86	ND	92.43	44.34	23.62	62.96
BBP	ND	73.41	67.28	3.11	53.57	ND	173.4	67.84	51.19	66.67
DBEP	95.09	641.4	226.9	74.59	100	44.95	851.7	374.8	63.32	100
DCHP	94.07	648.09	234.2	71.83	100	83.77	199.7	125.9	22.98	100
DEHP	45.41	599.6	189.1	70.33	100	31.89	180.0	85.98	28.43	100
DPhP	ND	42.19	37.89	4.15	50	ND	ND	NC	NC	0
DNOP	ND	53.65	51.38	2.13	60.71	ND	59.45	9.53	30.26	81.48
∑PAEs	1215	3014	1840	26.26		1384	3399	2176	26.63	

N: the number of sampling sites. CV: coefficient of variation. ND: lower than the detection limits. NC: not calculated.

## Data Availability

The data that support the findings of this study are available from the corresponding author, S.X. Liang (liangsx168@126.com), upon reasonable request.

## Supplementary Materials

The following are available online at https://www.mdpi.com/article/10.3390/ijerph20042918/s1, Table S1: Coordinates and classification of the sampling sites; Table S2: The linear coefficients, method detection limit (MDL), method quantification limit (MQL) and recovery for each individual PAEs; Table S3: Detailed parameter values of different exposure pathways; Table S4: Average concentrations of PAEs in the overlying water from sampling sites; Table S5: Toxicity data and correlative information of the selected PAE species exposed to the sensitive aquatic species; Table S6: Results of routine water quality index determination; Table S7: Variance interpretation rate and extraction factor load matrix after principal component analysis of overlying water; Figure S1: Effect of different elution solvents on the recovery; Figure S2: The compositions and proportions of PAEs detected in each sampling site in October (A) and May (B); Figure S3: Pearson correlation analysis between the individual PAE and conventional indices in October (A) and May (B).
