# Supplementary material for: Distribution, Source Apportionment and Risk Assessment of Phthalate Esters in the Overlying Water of Baiyang Lake, China
Source: Int J Environ Res Public Health. 2023 Feb 7;20(4):2918. doi: 10.3390/ijerph20042918 (PMC9957158; doi:10.3390/ijerph20042918)
Supplement: Supplementary file 1 [file ijerph-20-02918-s001.zip › ijerph-2124781-supplementary.pdf]

Supplementary Material

Distribution, source and risk assessment of phthalate esters in the overlying water of Baiyang Lake, China

Chang Liu [1,†], Liguo Fu [1,†], Hui Du [1], Yaxue Sun [1], Yihong Wu [2], Cheng Li [2], Jikun Tong [3], Shuxuan Liang [2,*]

[1] Key Laboratory of Hebei Provincial Analytical Science and Technology, College of Chemistry and Environmental Science, Hebei University, Baoding 071002, China.

[2] Hebei Provincial Academy of Ecological Environmental Science, Shijiazhuang 050037, China.

[3] Baiyangdian Watershed Ecological Environmental Monitoring Center, Baoding 071051, China.

1 Both authors contribute equally to this work.

*Corresponding author E-mail: liangsx168@126.com, Tel: +86-312-507-9359

Figure captions and Table legends

Fig. S1. Effect of different elution solvents on the recovery.

Fig. S2. The compositions and proportions of PAEs detected in each sampling site in October (A) and May (B).

Fig. S3. Pearson correlation analysis between the individual PAE and conventional indices in October (A) and May (B).

Table S1 Coordinates and classification of the sampling sites.

Table S2 The linear coefficients, method detection limit (MDL), method quantification limit (MQL) and recovery for each individual PAEs (n=16).

Table S3 Detailed parameter values of different exposure pathways.

Table S4 Average concentrations of PAEs in the overlying water from sampling sites.

Table S5 Toxicity data and correlative information of the selected PAE species exposed to the sensitive aquatic species.

Table S6 Results of routine water quality index determination.

Table S7 Variance interpretation rate and extraction factor load matrix after principal component analysis of overlying water.

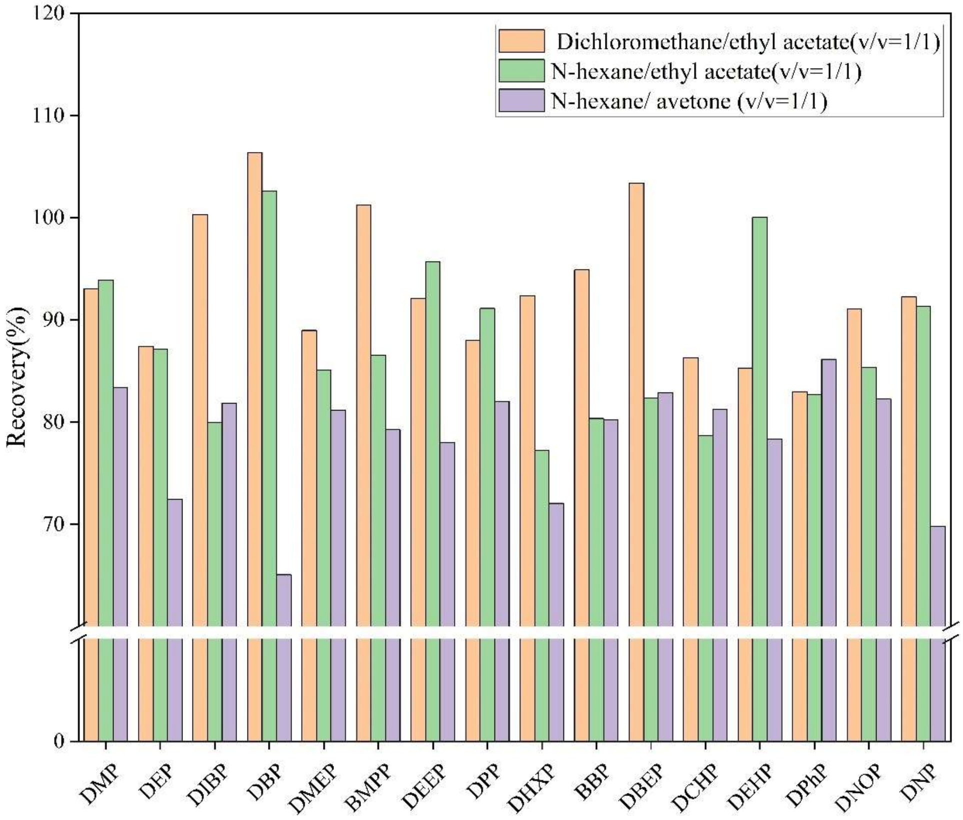

Figure S1. Effects of different elution solvents on the recovery.

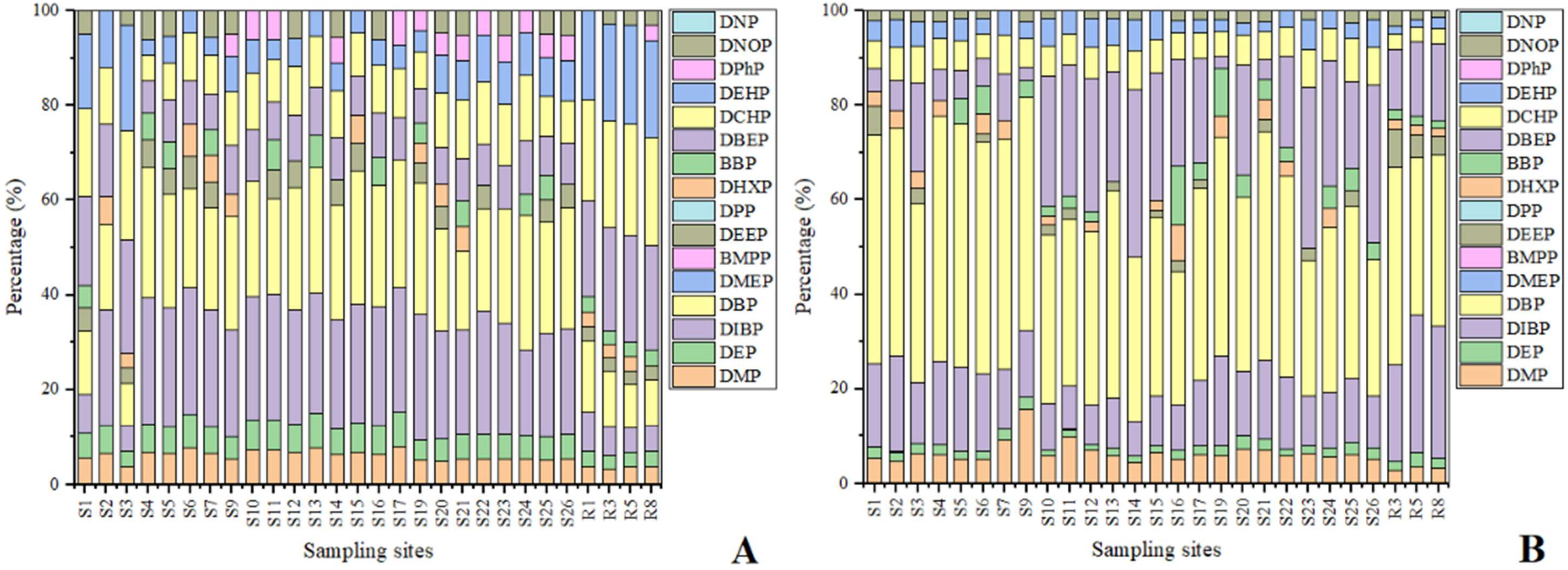

Figure S2. The compositions and proportions of PAEs detected in each sampling site in October (A) and May (B).

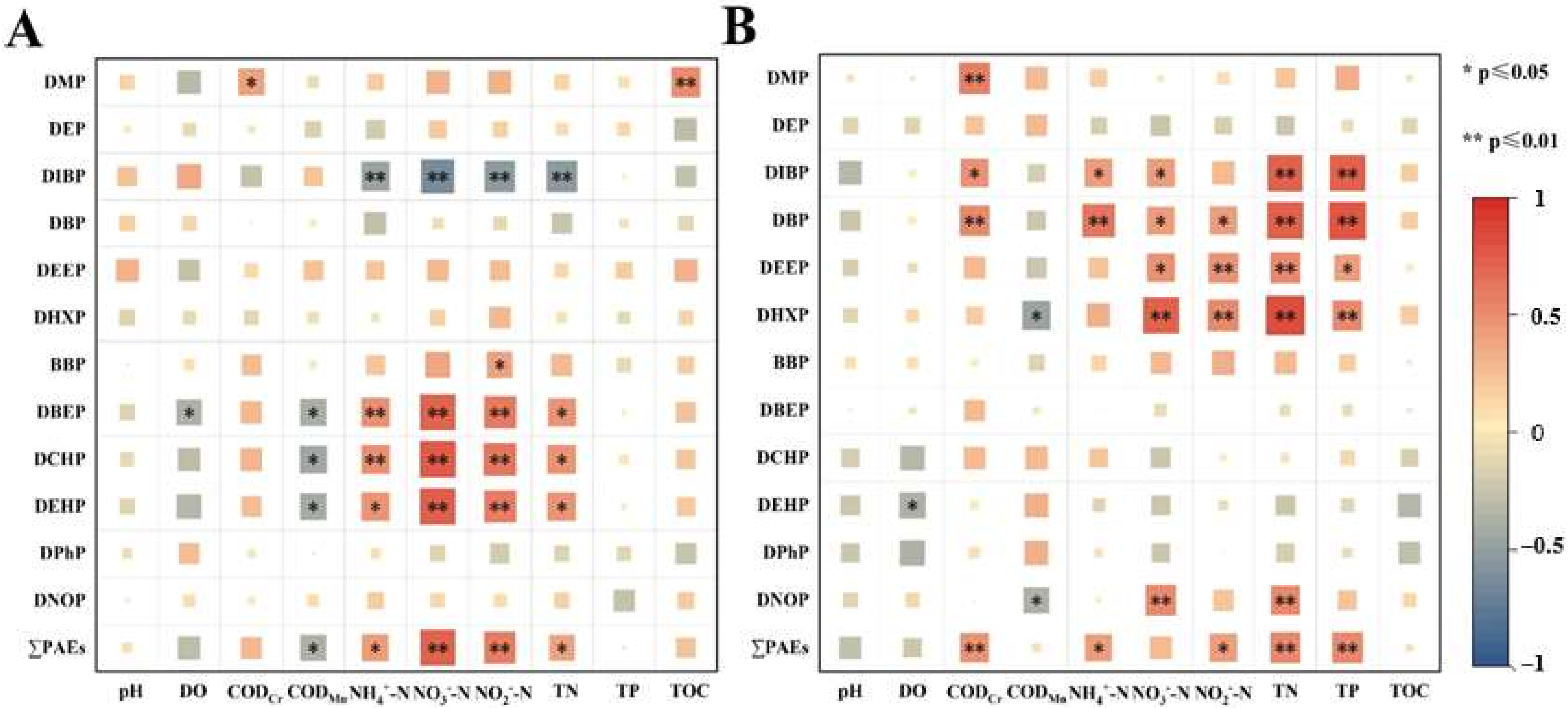

Figure S3. Pearson correlations analysis between the individual PAE and conventional indices in October (A) and May (B). **p means the correlation was significant when the confidence (bilateral) was 0.01. *p means the correlation was significant when the confidence (bilateral) was 0.05.

Table S1. Coordinates and classification of the sampling sites.

Sampling site	Longitude	Latitude	Region	Classification
S1	115.9347°E	38.9056°N		Rural area
S2	115.9931°E	38.9599°N		Natural area
S3	116.0312°E	38.0012°N		Rural area
S4	116.0312°E	38.9609°N		Rural area
S5	116.0257°E	38.8219°N		Rural area
S6	116.0889°E	38.8881°N		Rural area
S7	115.9519°E	38.8409°N		Rural area
S9	115.9946°E	38.9164°N		Rural area
S10	115.9931°E	38.8778°N		Natural area
S11	116.0510°E	38.8763°N		Natural area
S12	115.9877°E	38.8311°N		Natural area
S13	115.8819°E	38.8567°N	Baiyang	Natural area
S14	115.8943°E	38.8343°N	Lake	Rural area
S15	115.8819°E	38.8171°N		Natural area
S16	116.0222°E	38.8196°N		Natural area
S17	116.0046°E	38.9642°N		Natural area
S19	116.0719°E	38.9061°N		Natural area
S20	115.9639°E	38.9049°N		Rural area
S21	115.9714°E	38.9672°N		Natural area
S22	116.0149°E	38.7964°N		Rural area
S23	116.0081°E	38.8361°N		Natural area
S24	116.0674°E	38.8691°N		Natural area
S25	115.99697°E	38.8438°N		Natural area
S26	116.0109°E	38.9293°N		Rural area
R1	115.6698°E	38.8361°N	Fu River	Upstream
R3	115.8188°E	38.7468°N	Fu River	Upstream
R5	115.8443°E	38.8791°N	Xiaoyi River	Upstream
R8	115.7191°E	38.0836°N	Bao River	Upstream

Table S2. The linear coefficients, method detection limit (MDL), method quantification limit (MQL) and recovery for each individual PAEs.

PAEs	CAS Number	Linear regressing equation	R^2	MLD ($\mu g \cdot L^{-1}$)	MQL ($\mu g \cdot L^{-1}$)	Recovery (%)	RSD (%)
DMP	131–11–3	y=21281.1145x–2743.4217	0.9995	0.0082	0.0246	90.35	10.32
DEP	84–66–2	y=182541.7236x–3514.9538	0.9987	0.0041	0.0123	93.78	9.97
DIBP	84–69–5	y=197535.6234x–2502.8125	0.9983	0.0028	0.0084	101.73	8.84
DBP	84–74–2	y=232684.7121x–2788.8867	0.9982	0.0021	0.0063	98.57	9.03
DMEP	117–82–8	y=12903.8742x–623.3596	0.9975	0.0102	0.0306	84.91	8.73
BMPP	146–50–9	y=49532.5173x–1697.6425	0.9977	0.0091	0.0273	88.27	8.48
DEEP	605–54–9	y=19603.8992x–532.7969	0.9991	0.0121	0.0363	87.92	7.15
DPP	131–18–0	y=236758.2131x–13543.4778	0.9973	0.0133	0.0399	99.48	8.31
DHXP	84–75–3	y=121008.1211x–2798.9635	0.9978	0.0233	0.0699	109.35	6.99
BBP	85–68–7	y=61828.1642x–2473.3459	0.9980	0.0154	0.0462	100.02	9.56
DBEP	117–83–9	y=16338.9576x–4273.3321	0.9969	0.0132	0.0396	104.72	8.04
DCHP	84–61–7	y=118196.4498x–532.1504	0.9991	0.0155	0.0465	91.29	10.26
DEHP	117–81–7	y=97435.8336x–6775.6578	0.9988	0.0639	0.1917	90.66	9.97
DPhP	84–62–8	y=117332.4846x–7486.0839	0.9982	0.0472	0.1416	103.15	10.42
DNOP	117–84–0	y=8674.7641x–1236.0327	0.9971	0.0332	0.0996	108.74	8.89
DNP	84–76–4	y=69802.7577x–606.4246	0.9995	0.0298	0.0894	113.97	9.58

R^2 represents the correlation coefficient of PAEs linear regression equation.

Table S3. Detailed parameter values of different exposure pathways.

Abbreviation	Designation	Unit	Value		
CC	conversion coefficient	/	0.1		
RM	boiling residue ratio	/	0.1		
F	intestinal adsorption ratio	/	1		
FE	bathing frequency	$t\cdot d^{-1}$	0.3		
EF_{dri}	drinking exposure frequency	$d\cdot a^{-1}$	365		
EF_{der}	skin contact exposure frequency	$d\cdot a^{-1}$	350		
PC	skin permeability constant of chemical substance	$cm\cdot h^{-1}$	0.001		
MEC	pollutant concentration	$\mu g\cdot L^{-1}$	Table S4		
τ	residence time	h	1		
IR_w	daily drinking water volume	$L\cdot d^{-1}$	Male:1.825	Female:1.350	child:1.000
BW	body weight	kg	Male:64.8	Female:55.1	child: 26.8
SA	surface area of the skin	cm^2	Male:17000	Female:15000	child:8490
ED	exposure duration	a	Male:74	Female:78	child:12
AT	average exposure time	d	Male:27010	Female:28470	child:4380
TE	bath time	h	Male:0.40	Female:0.60	child:0.15
RfD	long-term intake reference dose	$mg\cdot(kg\cdot d)^{-1}$	DMP: 10<br>DBP: 0.1<br>DEHP: 0.02	DEP: 0.8<br>DHXP: 0.2<br>DNOP: 0.01	DIBP: 0.1<br>BBP: 0.2
SF	carcinogenic slope factors	$mg\cdot(kg\cdot d)^{-1}$	DEHP:0.014		

Table S4. Average concentrations of PAEs in the overlying water from sampling sites.

Sampling site	DMP (μg·L^{-1})		DEP (μg·L^{-1})		DIBP (μg·L^{-1})		DBP (μg·L^{-1})		DHXP (μg·L^{-1})		BBP (μg·L^{-1})		DEHP (μg·L^{-1})		DNOP (μg·L^{-1})	
	Oct	May	Oct	May	Oct	May	Oct	May	Oct	May	Oct	May	Oct	May	Oct	May
S1	0.096	0.118	0.084	0.048	0.140	0.385	0.226	1.053	ND	0.070	0.081	ND	0.266	0.090	0.082	0.046
S2	0.092	0.104	0.083	0.042	0.345	0.445	0.258	1.058	0.081	0.084	ND	ND	0.170	0.128	ND	0.041
S3	0.097	0.099	0.084	0.033	0.137	0.205	0.235	0.593	0.081	0.057	ND	ND	0.574	0.080	0.083	0.039
S4	0.093	0.099	0.084	0.035	0.376	0.294	0.387	0.859	ND	0.056	0.081	ND	0.045	0.061	0.086	0.038
S5	0.097	0.120	0.083	0.037	0.374	0.407	0.355	1.176	ND	ND	0.081	0.124	0.082	0.108	0.082	0.039
S6	0.093	0.117	0.083	0.034	0.318	0.368	0.246	1.100	0.081	0.091	ND	0.135	0.056	0.073	ND	0.039
S7	0.095	0.126	0.084	0.033	0.365	0.179	0.320	0.677	0.082	0.057	0.082	ND	0.058	0.072	0.083	ND
S9	0.094	0.256	0.085	0.039	0.395	0.231	0.422	0.799	0.082	ND	ND	0.055	0.135	0.056	0.087	0.038
S10	0.095	0.184	0.084	0.037	0.341	0.308	0.320	1.106	ND	0.061	ND	0.066	0.093	0.180	ND	0.053
S11	0.094	0.209	0.084	0.032	0.344	0.198	0.262	0.748	ND	ND	0.081	0.056	0.053	0.104	ND	ND
S12	0.096	0.209	0.085	0.037	0.349	0.257	0.372	1.106	ND	0.061	ND	0.066	0.085	0.180	0.085	0.053
S13	0.091	0.129	0.084	0.032	0.297	0.236	0.309	0.956	ND	ND	0.081	ND	0.064	0.123	ND	0.037
S14	0.098	0.085	0.085	0.030	0.356	0.144	0.375	0.684	ND	ND	ND	ND	0.090	0.130	0.086	0.036
S15	0.093	0.168	0.085	0.038	0.352	0.274	0.395	0.981	0.082	0.057	ND	ND	0.064	0.159	ND	ND
S16	0.090	0.077	0.084	0.029	0.353	0.148	0.360	0.426	ND	0.116	0.083	0.189	0.078	0.037	0.086	0.034
S17	0.090	0.118	0.083	0.034	0.299	0.271	0.308	0.789	ND	ND	ND	0.068	0.054	0.056	ND	0.036
S19	0.097	0.108	0.086	0.036	0.514	0.348	0.538	0.850	0.082	0.081	0.082	0.185	0.087	0.048	ND	0.034
S20	0.089	0.094	0.085	0.035	0.418	0.175	0.398	0.477	0.085	ND	ND	0.061	0.146	0.032	0.085	0.034
S21	0.088	0.100	0.085	0.034	0.366	0.242	0.276	0.698	0.085	0.060	0.089	0.062	0.137	0.032	0.086	0.034

Sampling site	DMP (μg·L^{-1})		DEP (μg·L^{-1})		DIBP (μg·L^{-1})		DBP (μg·L^{-1})		DHXP (μg·L^{-1})		BBP (μg·L^{-1})		DEHP (μg·L^{-1})		DNOP (μg·L^{-1})	
	Oct	May	Oct	May	Oct	May	Oct	May	Oct	May	Oct	May	Oct	May	Oct	May
S22	0.090	0.103	0.086	0.026	0.442	0.272	0.363	0.761	ND	0.055	ND	0.052	0.164	0.064	ND	ND
S23	0.089	0.118	0.085	0.033	0.381	0.200	0.397	0.539	ND	ND	ND	ND	0.148	0.117	0.087	0.038
S24	0.094	0.087	0.086	0.030	0.324	0.180	0.499	0.544	ND	0.064	0.081	0.070	0.160	0.060	ND	ND
S25	0.091	0.081	0.086	0.036	0.386	0.186	0.414	0.492	ND	ND	0.089	0.066	0.143	0.043	0.086	0.036
S26	0.088	0.095	0.085	0.046	0.371	0.210	0.423	0.548	ND	ND	ND	0.066	0.139	0.115	0.086	0.034
R1	0.097	NC	0.088	NC	0.223	NC	0.406	NC	0.081	NC	0.085	NC	0.571	NC	0.087	NC
R3	0.095	0.076	0.084	0.060	0.180	0.583	0.334	1.198	0.082	0.061	0.081	0.063	0.663	0.111	0.083	0.093
R5	0.099	0.110	0.083	0.088	0.144	0.898	0.247	1.026	0.081	0.060	0.081	0.058	0.574	0.051	0.082	0.058
R8	0.094	0.104	0.083	0.070	0.140	0.925	0.246	1.196	ND	0.058	0.081	0.057	0.521	0.074	0.082	0.049

ND means lower than the limits of detection.

NC means the samples were not collected.

Table S5. Toxicity data and correlative information of the selected PAE species exposed to the sensitive aquatic species.

PAEs	Population	Species	Toxicity data ($\mu g \cdot L^{-1}$)	AF	$PNEC_{water}$ ($\mu g \cdot L^{-1}$)
DMP	Algae	*Pseudokirchneriella subcapitata*	4 d, population, *NOEC* = 10,000	10	1,000
	Crustacean	*Daphnia magna*	21 d, mortality, *NOEC* = 9,600	10	960.0
	Fish	*Oncorhynchus mykiss*	102 d, mortality, *NOEC* = 11,000	10	1,100
DEP	Algae	*Pseudokirchneriella subcapitata*	96 h, population, *NOEC* = 8,106	10	810.6
	Crustacean	*Americamysis bahia*	21 d, mortality, *NOEC* = 2,700	10	270.0
	Fish	*Lepomis macrochirus*	28 d, morphology, *NOEC* = 1,650	10	165.0
DBP	Algae	*Pseudokirchneriella subcapitata*	96 h, population, *NOEC* = 210	10	21.00
	Crustacean	*Americamysis bahia*	21 d, mortality, *NOEC* = 260	10	26.00
	Fish	*Oncorhynchus mykiss*	99 d, growth, *NOEC* = 100	10	10.00
BBP	Algae	*Pseudokirchneriella subcapitata*	4 d, population, *NOEC* = 60	10	10.00
	Crustacean	*Daphnia magna*	21 d, mortality, *NOEC* = 760	10	10.00
	Fish	*Oncorhynchus mykiss*	124 d, mortality, *NOEC* = 200	10	10.00
DEHP	Algae	*Pseudokirchneriella subcapitata*	4 d, population, EC_{50} = 100	1,000	0.10
	Crustacean	*Mytilus edulis*	21 d, mortality, *NOEC* = 42	50	0.84
	Fish	*Gasterosteus aculeatus*	28 d, mortality, *NOEC* = 300	50	6.00
DIBP	Crustacean	*Nitocra spinipes*	4 d, mortality, LC_{50} = 3,000	10	300.0

	Fish	*Pimephales promelas*	96 h, mortality, LC_{50} = 900	1,000	0.90
DnOP	Fish	*Fathead Minnow*	28 d, mortality, *NOEC* = 8,300	10	830.0

Table S6. Results of routine water quality index determination.

Unit	October				May			
	Concentration ($mg \cdot L^{-1}$)			CV (%)	Concentration ($mg \cdot L^{-1}$)			CV (%)
	Min	Max	Mean		Min	Max	Mean	
pH	7.5	8.9	8.2	5.45	7.8	8.9	8.4	2.41
DO	5.5	9.5	7.9	10.78	6.2	14.2	9.5	18.86
COD_{Mn}	4.75	10.25	8.33	19.44	3.94	7.34	6.47	13.02
COD_{Cr}	12.04	62.46	29.79	49.89	22.66	64.3	36.86	22.66
NO_2^--N	0.012	0.073	0.028	52.65	0.009	0.104	0.041	51.13
NO_3^--N	0.105	4.792	0.913	114.72	0.007	2.501	0.439	141.98
NH_4^+-N	0.42	1.82	0.34	88.28	0.16	2.00	0.43	79.45
TN	1.52	7.24	2.67	44.04	0.31	3.41	1.09	76.11
TP	0.004	0.157	0.048	64.74	0.018	0.434	0.057	145.83
TOC	5.18	18.03	8.41	28.35	5.34	28.21	9.64	42.34

CV means coefficient of variation.

Table S7. Variance interpretation rate and extraction factor load matrix after principal component analysis of overlying water.

PAEs	Component matrix in October				Component matrix in May		
	PC1	PC2	PC3	PC4	PC1	PC2	PC3
DMP	0.287	-0.034	0.757	-0.093	-0.123	0.637	0.043
DEP	0.074	0.894	0.047	-0.145	0.914	-0.095	-0.043
DIBP	-0.777	0.49	-0.175	-0.103	0.907	-0.05	0.171
DBP	-0.302	0.862	0.042	0.01	0.671	0.472	0.340
DEEP	0.143	0.138	0.701	0.128	0.866	-0.136	-0.08
DHXP	0.336	0.046	0.217	-0.66	0.198	-0.033	0.774
BBP	0.135	0.034	0.460	0.414	-0.065	-0.233	0.709
DBEP	0.960	-0.085	0.242	-0.004	0.108	0.654	-0.398
DCHP	0.977	-0.015	0.182	0.007	-0.073	0.937	-0.150
DEHP	0.975	-0.034	0.188	0.020	-0.027	0.961	-0.148
DPhP	-0.092	0.507	-0.636	0.252	NC	NC	NC
DNOP	0.207	-0.073	0.124	0.681	0.738	-0.008	0.045
Variance interpretation rate of PC (%)	38.938	15.513	10.574	9.845	32.564	27.939	11.179
Cumulative variance interpretation rate of PCs (%)	38.938	54.451	65.025	74.870	32.564	60.503	71.863

PC means the primary component was distilled to govern above-mentioned PAEs by the primary component analysis.

NC means not calculated.
